# Triple Arterial Minimally Invasive Direct Coronary Artery Bypass Grafting: Step-By-Step Technique Report

**DOI:** 10.21470/1678-9741-2024-0193

**Published:** 2025-05-20

**Authors:** Danko Grujic, Vojkan Aleksic, Tatjana Gazibara, Vladimir Milicevic, Radmila Karan

**Affiliations:** 1 Department of Cardiac Surgery, University Clinical Center of Serbia, Belgrade, Serbia; 2 Institute of Epidemiology, Faculty of Medicine, University of Belgrade, Belgrade, Serbia; 3 Department of Anesthesiology, University Clinical Center of Serbia, Belgrade, Serbia; 4 Faculty of Medicine, University of Belgrade, Belgrade, Serbia

**Keywords:** Coronary Artery Bypass, Coronary Artery Disease, Surgical Anastomosis, Mammary Arteries, Radial Artery

## Abstract

Minimally invasive direct coronary artery bypass grafting (MIDCAB) has considerable benefits over the conventional coronary artery bypass grafting procedure. This case report presents the MIDCAB procedure in a multivessel coronary disease using triple arterial grafts and four arterial anastomoses. The initial anastomosis was made between the left intrathoracic mammary artery (LIMA) and the radial artery (RA), as an end-to-side "T" graft. Next, the RIMA was used to left anterior descending anastomosis. The first obtuse marginal (OM1) branch was grafted to allow LIMA-OM1 side-to-side anastomosis. Then, with the diagonal branch (Dg) opened, the formation of a "jumping" anastomosis was made using LIMA-OM1-Dg. The posterior descending artery (PDA) was used to create a LIMA-RA-PDA.

## INTRODUCTION

**Table:** 

Abbreviations, Acronyms & Symbols	
CPB	= Cardiopulmonary bypass
DG	= Diagonal branch
LAD	= Left anterior descending
LIMA	= Left intrathoracic mammary artery
MIDCAB	= Minimally invasive direct coronary artery bypass grafting
OM	= Obtuse marginal
PDA	= Posterior descending artery
RA	= Radial artery
RCA	= Right coronary artery
RIMA	= Right intrathoracic mammary artery

Minimally invasive direct coronary artery bypass grafting (MIDCAB) involves a small anterior left thoracotomy incision of 4-6 cm. Because of this, there are considerable benefits over the conventional coronary artery bypass grafting procedure through full sternotomy: smaller incisions, reduction in hospital stay, faster recovery, and lower risk of bleeding and wound infectious^[1]^. Nevertheless, a steep learning curve and the complexity of performing MIDCAB prevent it from becoming a routine procedure worldwide^[2]^. Typically, MIDCAB procedure uses the left intrathoracic mammary artery (LIMA) as a bypass graft for the left anterior descending (LAD) artery in patients with single-vessel coronary LAD artery disease^[3]^. A few MIDCAB procedures include two arterial grafts, and MIDCAB performed on a multivessel coronary disease with three arterial grafts (four anastomoses) without cardiopulmonary bypass (CPB) (aortic non-touch) technique is rare^[4]^.

## CASE PRESENTATION

In this case report, we present the MIDCAB procedure in a multivessel coronary disease using triple arterial grafts, to achieve complete revascularization of myocardium. Approval to conduct the study was granted by the Ethics Committee of the University Clinical Center of Serbia (approval no. 936/19; issued on February 29, 2024). The patient provided a signed informed consent to have his data presented in this report.

A 63-year-old man was referred to our institution for surgical revascularization of myocardium from a secondary health care center. Previously he had chest pain during exertion and went to the hospital where coronary angiography was performed. On coronary angiography, the three-vessel coronary disease was diagnosed with 80% LAD proximal stenosis, 60% obtuse marginal (OM) stenosis, 90% right coronary artery (RCA) stenosis, and 80-90% posterior descending artery (PDA) stenosis. The patient had a history of hypertension and dyslipidemia and no other diagnosed chronic illnesses.

### Surgical Technique

Overall, the patient was hemodynamically stable. On echocardiography, the left ventricular ejection fraction was estimated at 50%, the left ventricular end-diastole diameter was 53 mm, and the end-systole diameter was 40 mm. Based on preoperative testing, the patient was scheduled for an elective cardiac surgery. After preoperative preparation, the patient was positioned at the right side and was intubated with a double-lumen endotracheal tube, which allows selective lung ventilation. Under general anesthesia, the main incision (of around 5 cm in length) was performed at the fourth intercostal space just below the left nipple thereby opening pleural cavity where thoracic retractor was placed. This incision was used to perform MIDCAB. Then, it was placed the MIDCAB retractor for harvesting the internal mammary artery. At the same time, two auxiliary incisions were made: 1) a subxiphoid incision (1 cm) to place the xiphoid blade part of the retractor which would allow access to the right intrathoracic mammary artery (RIMA) and 2) a secondary incision near the main incision to place the laparoscopic access port for the harmonic scalpel.

**Fig. 1:**
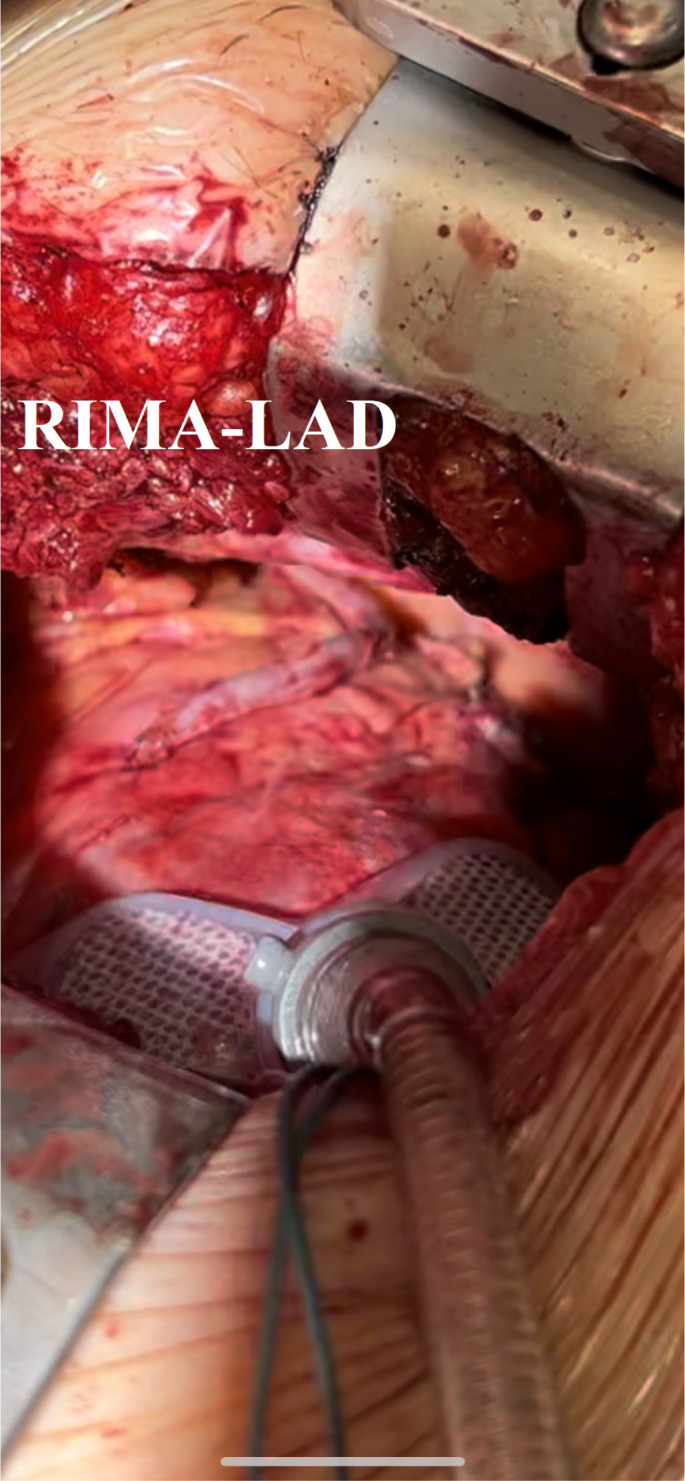
Anastomosis of right intrathoracic mammary artery (RIMA) to left anterior descending (LAD) coronary artery.

The RIMA and the LIMA were harvested using harmonic scalpel hook-type knives from the first all the way to the sixth rib. Proximal LIMA preparation requires a total short apnea phase. Following heparinization (100-150 IU/kg), distal dissection of RIMA and LIMA was performed and covered with papaverine to optimize blood flow through the grafts. In a parallel guided act on the non-dominant arm, a radial artery (RA) was harvested using a “fully no-touch technique” harmonic scalpel^[5]^. The initial anastomosis was made between LIMA and RA, as an end-to-side "T" graft. Next, the RIMA was used as an in situ graft to LAD anastomosis (Figure 1), which was described in previous studies as a safe graft option^[6]^.

The suction stabilizer starfish was placed on top of the heart, and the first OM (OM1) branch of the circumflex artery was identified and positioned for grafting to allow for the making of the LIMA-OM1 side-to-side anastomosis. Then, the heart was positioned with suction stabilizer octopus to open the diagonal branch (Dg) where the formation of a "jumping" anastomosis was made using LIMA-OM1-Dg. The PDA, branch of the RCA, was also identified and used to make a LIMA-RA-PDA formation. The position for the PDA was formed by suction stabilizers octopus.

A pericardial drain was inserted through the orifice created for the port, at the end of the MIDCAB procedure. All anastomoses were performed off pump (Video 1). The surgical wound was closed in layers, and on the fourth postoperative day, the patient was discharged from the hospital. Two months after the operation, on the control re-coronarography, all grafts showed normal flow.

**Video 1:**
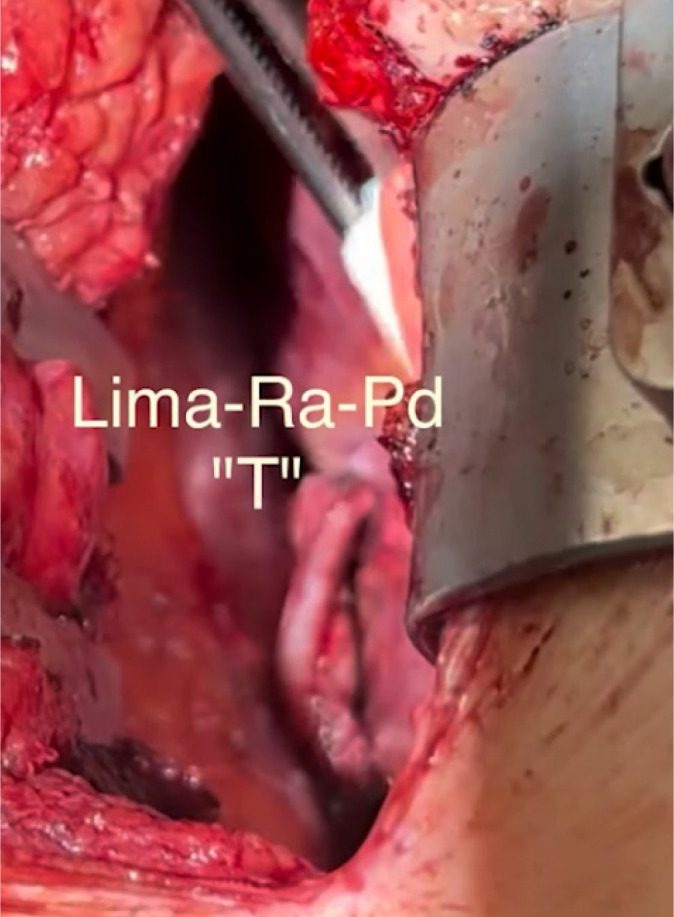
Video showing a whole procedure of total arterial coronary anastomoses through minimally invasive thoracotomy. Link: https://youtu.be/MyTe0JuL_Wc

## DISCUSSION

This is seldom a MIDCAB operative technique considering it involves three arterial bypass grafts without CPB. Our case demonstrates a surgical technique that includes complete revascularization in a patient with multivessel coronary artery disease. The entire procedure was performed without manipulating the ascending aorta, thereby decreasing the risk of stroke. In addition, it overcomes the challenge of selecting a patient with an isolated single vessel coronary disease. In fact, patients also prefer this option when having multivessel coronary disease. More importantly, a complete surgical revascularization of the myocardium is achieved with arterial grafts, which allows for optimum long-term postoperative outcomes.

## CONCLUSION

Using this minimally invasive technique, postoperative morbidity could potentially be reduced. This procedure demands a learning curve and sophisticated educational concept.

**Table:** 

Authors’ Roles & Responsibilities	
DG	Substantial contributions to the conception or design of the work; and the interpretation of data for the work; drafting the work and revising it critically for important intellectual content; agreement to be accountable for all aspects of the work in ensuring that questions related to the accuracy or integrity of any part of the work are appropriately investigated and resolved; final approval of the version to be published
VA	Substantial contributions to the conception or design of the work; drafting the work; agreement to be accountable for all aspects of the work in ensuring that questions related to the accuracy or integrity of any part of the work are appropriately investigated and resolved; final approval of the version to be published
TA	Substantial contributions to the conception or design of the work; and the analysis of data for the work; revising the work; final approval of the version to be published
VM	Substantial contributions to the conception or design of the work; revising it critically for important intellectual content; final approval of the version to be published
RK	Substantial contributions to the conception or design of the work; revising it critically for important intellectual content; final approval of the version to be published
